# Intestinal Obstruction1Read at a meeting of the Eighth District Branch of the Medical Society of the State of New York, held at Buffalo, September 25 and 26, 1908.

**Published:** 1908-09

**Authors:** W. H. Loughhead

**Affiliations:** Andover, N. Y.


### Intestinal Obstruction.[1]

BY W. H. LOUGHHEAD, M. D., Andover, N. Y.

UNDER this term are included all those cases in which the contents of the intestinal canal are obstructed in their onward passage by causes or conditions arising within the abdomen or pelvis. Before a knowledge of pathology existed, the term was applied indiscriminately to all cases presenting the well marked indications of sufficient obstruction to the onward flow of the intestinal contents. Thus, with advancing pathological observations, definite classes have been made having a distinctive anatomical basis, and to which special designation have been given.

At the present day with a case before us in which we recognise the existence of an obstruction, we endeavor to go still further, to ascertain as far as possible, the location, the cause, and the nature of the trouble. To accomplish this in some cases is not difficult; in others it involves the highest knowledge and acumen, and in not a few is really impossible. A complete or partial occlusion of any portion of the intestinal canal may occur suddenly, causing acute obstruction; or be of gradual devel-

1. Read at a meeting of the Eighth District Branch of the Medical Society of the State of New York, held at Buffalo, September 25 and 26, 1908.

opment and thus reduce the diameter of the intestinal canal, causing chronic obstruction.

An acute obstruction may become chronic and a chronic case may become acute; therefore, it is sometimes difficult to determine accurately the condition with which we have to deal, as one form may take on some or many of the symptoms of the other. We do not purpose in this paper to discuss all the different classifications of intestinal obstruction but to call attention for a few moments to the report of a case which occurred at Andover, N. Y., in December, 1906.

I was called to the residence of Mr. R., December 19, about 2 p. m., to see his mother, she being a lady 65 years of age, the mother of four children, the oldest 43 years and the youngest 33 years. There was a negative family history. For some time she had been troubled with constipation, and at 4 o'clock in the morning she was suddenly attacked with severe abdominal pain which caused intense distress and continued. The pains were at first paroxysmal, and then after a time continuous, the suffering increasing as the case continued.

Nausea was followed by vomiting, which increased in severity until it constituted a most distressing symptom. The contents of the stomach were first voided; this was followed by a greenish fluid stained with bile, which became brown, and finally stercoraceous.

I gave her a hypodermic injection of heroin 1-12 gr. to relieve the pain. Saline and other cathartics, a high enema was also given to remove all fecal substance from the lower bowel, the entire colon being flushed, one gallon of water being used containing two ounces of magnesium sulphate, and one ounce of oleum terebinthiæ, but without results. The general symptoms indicated the grave character of the disease. The features were pinched, the surface cold and clamy, temperature being subnormal. The thirst was marked and the mouth and tongue were dry. The pulse was rapid, feeble, and at times irregular, the breathing being correspondingly more frequent.

There was suppression of urine. There was some tenderness upon pressure and a slight distention which involved the center of the abdomen rather than the sides. With the continued pain, subnormal temperature, rapid and feeble pulse, with early and continued vomiting, and obstinate constipation, I diagnosticated an obstruction of the small intestines.

Intestinal obstruction is a surgical ailment and can only be adequately treated by surgical operation. The facilities for operting at the house were poor, hence the patient was placed upon a cot-bed and carried by train a distance of twenty miles to Saint James's Mercy Hospital, Hornell. Dr. Harvey P. Jack, of Canisteo, being informed by telephone that we desired an operation was in readiness to proceed when we arrived at the hospital, about 3 p. m. on the following day.

The patient having been made ready he proceeded to operate. An incision was made in the middle line of the abdomen below the umbilicus about four inches in length, not allowing any intestine to escape. He examined first with two fingers, then with the whole hand the right iliac region, bringing into view a portion of the small intestine containing an oval solid mass, which completely impacted the lumen of the intestine. Carefully protecting the abdominal cavity by packing gauze about the opening, and making a longitudinal incision in the intestine, he removed the mass which proved to be an enterolith, and thus relieved the obstruction. He then closed the opening in the intestine by the Lembert suture, replacing the intestine within the abdomen. He then proceeded to close the abdominal incision with proper drainage and dressing. The patient made a quick and uninterrupted recovery and is enjoying good health at the present time.

If a patient has taken cathartics or injections without results, if he is vomiting, has paroxysmal pain, and the intestines knot up and show themselves in ridges against the abdominal wall, the chances are nineteen out of twenty that there is either a complete or partial obstruction. Thus partial obstruction soon develops into a complete obstruction, by reason of the infection at the point of obstruction. Hence a patient exhibiting this train of symptoms should be treated by the physician long enough only to satisfy himself that the bowels will not move.

That knowledge may be obtained in from six to twelve hours, and from that time on the attendant is blamed for every hour that is lost before operation. And that is the sole object of this paper, to bring the attendant physician to a realisation of the need of immediate action in that he permit no delay to bring harm to his patient, and that he make an early diagnosis of obstruction.

